# Case report: Urolithiasis, nephrolithiasis and a urinary bladder malformation in a seven-month-old alpaca cria

**DOI:** 10.3389/fvets.2022.1038642

**Published:** 2022-12-13

**Authors:** Johannes Schregel, Sven Kleinschmidt, Alexandra von Altrock, Doris Hoeltig, Martin Ganter, Matthias G. Wagener

**Affiliations:** ^1^Clinic for Swine and Small Ruminants, Forensic Medicine and Ambulatory Service, University of Veterinary Medicine Hannover, Foundation, Hanover, Germany; ^2^Lower Saxony State Office for Consumer Protection and Food Safety, Food and Veterinary Institute Braunschweig/Hannover, Hanover, Germany; ^3^Clinic for Ruminants and Swine, Department of Veterinary Medicine, Freie Universität Berlin, Berlin, Germany

**Keywords:** South American camelid, azotemia, urinary dysfunction, ultrasound, kidney function analysis, urolithiasis, nephrolithiasis

## Abstract

Urolithiasis is a common condition in male small ruminants where predisposing factors have been identified. Occasionally, urolithiasis is diagnosed in South American camelids (SACs). However, nephrolithiasis is rarely diagnosed in ruminants. To our knowledge, this is the first report focusing on a combined appearance of nephrolithiasis and urolithiasis in an alpaca cria. A 7-month-old alpaca cria suffering from impaired urinary flow was presented for examination. On admission, the alpaca had a wet prepuce and showed a standing posture with a wide-based stance. Ultrasonographic examination of the abdomen showed a distended bladder. Clinical chemistry revealed azotemia and hypophosphatemia. After the first examination, repeated urination was observed. Conservative therapy using antibiotics, anti-inflammatory and spasmolytic drugs was started with the suspected diagnosis of urinary calculus. During the first 24 h, plasma concentrations of creatinine and urea decreased, but increased again during the following days. During the second day after admission, urination was not observed for 16 h while the concentration of urea and creatinine further increased. Therefore, the animal was euthanized due to financial concerns of the owner. Necropsy revealed that calculi were located in the left kidney as well as in the urethra. In addition, the animal exhibited uroperitoneum. The urinary bladder was intact, moderately distended with urine and showed a malformation, which was covered with a translucent mucosal membrane. Histologic examination revealed that this malformation was a bladder diverticulum. The extent to which the unilateral nephroliths affected the general condition and renal function of the animal is unclear, since the uroliths also cause azotemia, and abdominal pain. Further studies are needed for a better understanding of obstructive urinary disease in SACs.

## Introduction

Urolithiasis has been investigated intensively in the past decades in goats and sheep (1–3). Several disposing factors, including gender, age, castration status, and body condition score have been identified as risk factors for the formation of urolithiasis in these species (1, 2, 4, 5). However, the situation in South American camelids (SAC) has not been examined as closely. In a previous study, 34 cases of urolithiasis in SAC were analyzed retrospectively, the study classified castrated males as a potential risk group as well (6). Furthermore, it is discussed that the low water requirements related to metabolic body weight of SAC (7) might favor the formation of uroliths (8). Feeding also influences the formation of calculi. Comprehensive epidemiological studies on the chemical composition of uroliths in SACs are not available. Nonetheless, in a previous case of obstructive urolithiasis in a castrated male llama, silicate calculi were identified as the cause of obstruction in the urethra (9). In small ruminants, combined calculi consisting of amorphous magnesium calcium phosphate and struvite were most frequently detected, followed by calcium carbonate calculi (10). It is known that a diet high in concentrated feed predisposes the formation of struvite crystals in small ruminants (11). Moreover, overfeeding with calcium or vitamin D_3_ deficiency are discussed as possible predisposing factors for calculus formation due to elevated renal calcium excretion (12). Unlike uroliths, nephroliths are rarely diagnosed in ruminants. However, it is reported that in sheep, renal calculi formation depends on the location of pasture (13) and the geologica nature of the soil. Based on the few documented cases of nephrolithiasis in SAC, it is unclear whether unilateral nephrolithiasis leads to an increase in urinary substances in plasma or whether the contralateral kidney can compensate the malfunction (14). Since to our knowledge, there are no descriptions of nephrolithiasis in alpacas, we present the clinical, laboratory, and pathologic findings of an alpaca with urolithiasis, nephrolithiasis, and a bladder malformation.

## Case description

On January 10, 2022 (day 0), a 7-month-old alpaca cria with a body weight (BW) of 28.3 kg was presented to the clinic. The animal was weaned 4 weeks prior to admission. After weaning, the cria consumed only small amounts of solid food. The owners had noticed beginning signs of emaciation 1 week before admission. The cria was fed mainly hay or grass. Additionally, it received a small amount (50 g) of mineralized concentrate (Alpaca pacos, Eilers Futtermittel GmbH & Co. KG, Saerbeck, Germany) and a small piece of forage beet was fed, roughly chopped and soaked every day. The owners further reported that the cria could only pass urine in a dribbling manner in combination with intensified abdominal pressure. Before the alpaca was presented to the clinic, it had been treated with a non-steroidal antiphlogistic drug and with an antibiotic by the local veterinarian. Nine months prior to the admission to the clinic, another male cria had died on the farm at the age of 9 months and a necropsy had been performed. Pathologic examination of that male alpaca showed that both kidneys were swollen, had multifocal signs of hemorrhages, tubulus degeneration, and tubular necrosis.

### Clinical examination

At the general examination of the cria, the animal's coat was wet and dirty over a large area, especially in the ventral abdomen and hind limbs. Both hind limbs were splayed out. Heart rate was 56 beats per min, respiratory rate was 28 breaths per min, rectal temperature was 37.8°C, and body condition score was 2.5 of 5 (15), which were all within the reference limits. Prepositioning of the penis from the prepuce was not possible. Sonographic examination of the abdomen showed a severely filled urinary bladder with a diameter of 7.5 cm. Moreover, the urinary bladder was partially filled with hyperechogenic gravel. Sonographic examination indicated that no free fluid was present in the abdominal cavity. In the X-ray examination of the abdomen, the urinary bladder could not be precisely depicted. There were no indications of foreign bodies that could explain an obstruction of the urinary flow.

### Laboratory results

Blood samples were taken from the jugular vein (EDTA, Monovette 9 mL K3E, Lithium-Heparin, Monovette 9 mL LH and serum, Monovette 9 mL Z; all from Sarstedt AG & Co. KG, Nümbrecht, Germany) for laboratory tests). Furthermore, a serum sample was sent to an external laboratory (SYNLAB vet, Leipzig, Germany) for analysis of Vitamin D (25-OH) concentration. Feces were sampled for parasitological examination and investigated as previously reported (16). Laboratory diagnostics revealed azotemia, hypophosphatemia, and hypercalcemia (17) (Table 1). The hematological examination revealed slight anemia and mild neutrophilia with band neutrophils (Table 2). Erythrocyte and leukocyte abnormalities were not detected in the blood smear. Furthermore, parasitological investigation revealed a large number of coccidial oocysts.

**Table 1:** Concentration of various plasma parameters and urinary bladder diameter during the clinic stay.

**Sampling time**	**Admission**	**Day 1**	**Day 2 morning**	**Day 2 evening**	**Day 3**	**Reference value^a^**
Creatinine [μmol/l]	324	207	416	438	490	91.0–162
Urea [mmol/l]	19.8	18.8	23.5	25.2	25.7	3.2–9.1
Sodium[mmol/l]		144.6	145.9	142.7	138.3	138–159
Potassium[mmol/l]		5.62	4.15	4.26	4.75	4.4–6.1
Phosphate [mmol/l]	0.44	0.54	0.40	0.62	0.84	1.78–3.96
Magnesium [mmol/l]	0.79					0.8–1.18
Calcium [mmol/l]	2.74					2.1–2.7
Diameter urinary bladder [cm]	7.5		6	6.5	8.0	

a Reference values published by (17). Values for adult alpacas were referenced. For missing boxes, no data were available.

**Table 2:** Hematologic parameters at the time of admission (day 0) to the clinic.

**Parameter**		**Reference values^a^**
PCV^b^ [l/l]	0.21	0.29–0.37
Hemoglobin [mmol/l]	5.83	7.9–10.3
Leukocytes [10^9^/l]	11.1	9.8–15.8
Band neutrophils [10^9^/l]	0.33	0–0.1
Segmented neutrophils [10^9^/l]	9.32	4.5–9.3
Eosinophil granulocytes [10^9^/l]	0	1–3.6
Basophil granulocytes [10^9^/l]	0	0–0.3

a Reference values published by Hengrave Burri, Tschudi (18).

b PCV, packed cell volume.

### Treatment and further development

The cria was treated with a combination of metamizole (50 mg/kg BW i.v.) and butylscolpamin (0.4 mg/kg BW i.v. Boehringer Ingelheim Vetmedica GmbH, Ingelheim, Germany). Shortly after admission, defecation and urination were observed.

#### Day 1

On the first day after admission, the animal's urinary bladder was again ultrasounded and found to be heavily distended. The urinary bladder was aspirated under ultrasonographic control in the regio inguinalis and 60 ml of urine was sampled for further diagnostics in a plastic syringe (Omnifix, B. Braun Vet Care, B. Braun Vet Care GmbH, Melsungen, Germany). For this procedure, the regio inguinalis was shaved, and the punctured site was prepared using 70% ethanol as well as alcoholic iodine solution. The bladder was aspirated using a Veress needle. Simultaneously to the urine sample, blood was sampled from the *V. jugularis* (Lithium-Heparin) for kidney function analysis. The alpaca was treated again with a combination of metamizole (50 mg/kg BW i.v.), butylscolpamin (0.4 mg/kg BW i.v. Buscopan, Boehringer Ingelheim Vetmedica GmbH), and in addition to the previous treatment, with amoxicillin-sodium (10 mg/kg BW i.v., Selectavet Dr. Otto Fischer GmbH, Weyarn-Holzolling, Germany), omeprazole dissolved in 6 ml of sterile isotonic saline solution (1 mg/kg BW i.v. Hexal AG, Holzkirchen, Germany), and once with toltrazuril (20 mg/kg BW per os, Vibrac Tierarzneimittel GmbH, Bad Oldesloe, Germany). Moreover, oral substitution with highly digestible phosphate formulation (20 ml) (Veyx-Pharma GmbH, Schwarzenborn, Germany) and intravenous fluid therapy using 500 ml osmolar electrolyte solution (60 ml/h) (B. Braun Vet Care GmbH) was started. Urination and defecation were still observed, whereas feed intake was not present. The urine sample revealed glucosuria (10 g/l). Leukocytes were detected in the urine sediment. At day one following admission, plasma concentrations of creatinine, urea, glucose, and potassium in plasma were above reference limits (17). However, the concentration of creatinine and urea in plasma decreased compared to the initial sample (Table 1), whereas the concentration of phosphate was still below the reference limits. Kidney function analysis (19, 20) revealed that fractional excretion rates of glucose, phosphate, and water *via* urine were increased. The ratio of gamma-glutamyl-transferase (GGT) to creatinine in urine was elevated as well compared to reference limits in horses (19), indicating disintegration of tubular epithelial cells.

#### Day 2

Two days following admission, the concentrations of creatinine, urea, potassium, phosphate, and sodium in plasma were examined again and the aforementioned therapy was continued. The cria did not stand up voluntarily and spent most of the time lying in sternal recumbency. To monitor the urine excretion, a clean towel was taped around the abdomen of the cria. Repeated clinical chemistry examination revealed a further increase in azotemia (Table 1).

After 4 h, only a small amount of urination was documented. The pelvic cavity was examined again by using ultrasound. The size of the bladder had decreased to 4 cm x 6 cm. The owner was informed about the findings and therapy options were discussed. The owner did not agree to a diagnostic laparotomy to exclude urolithiasis as a potential reason for urethral obstruction. Therefore, the non-surgical therapy was proceeded. Additionally, the alpaca received dexamethasone (0.15 mg/kg BW i.v. Bela-Pharm GmbH &. Co. KG, Vechta, Germany). Intensive infusion therapy using two liters of isotonic saline solution (250 ml/h) (WDT—Wirtschaftsgenossenschaft Deutscher Tierärzte eG Garbsen, Germany) was started. Urination was continuously controlled and the bladder of the patient was examined at short intervals (2 h) with ultrasonography. Due to the infusion, the animal's general condition temporarily improved and it was able to stand up on its own. Six hours after the start of intensive fluid therapy, only a small volume of urine had been excreted, while the size of the bladder had increased to 6.5 x 6.5 cm. Plasma levels of creatinine and urea were measured again (Table 1). After 8 h without significant urination, infusion therapy was stopped. Ultrasonographic examination showed that the size of the bladder had increased to 7.5 x 7.5 cm.

#### Day 3

Three days following admission, the general condition of the animal deteriorated. Again, the cria refused to stand up voluntarily and remained in sternal recumbency. After 16 h without significant urination, ultrasonographic examination of the bladder was repeated and the diameter of the bladder had increased to 8 cm x 8 cm, while no further urine had been excreted. In parallel, the taken blood samples showed that azotemia levels had risen consistently (Table 1). In consultation with the owner, the animal was euthanized and sent for necropsy to the Food and Veterinary Institute Braunschweig/ Hannover, Germany. In retrospect, it turned out that the cria also suffered from Vitamin D (25-OH) deficiency [measured value 19. nmol/l reference value (21) 39–437 nmol/l].

### Gross pathology

**Figure 1:**
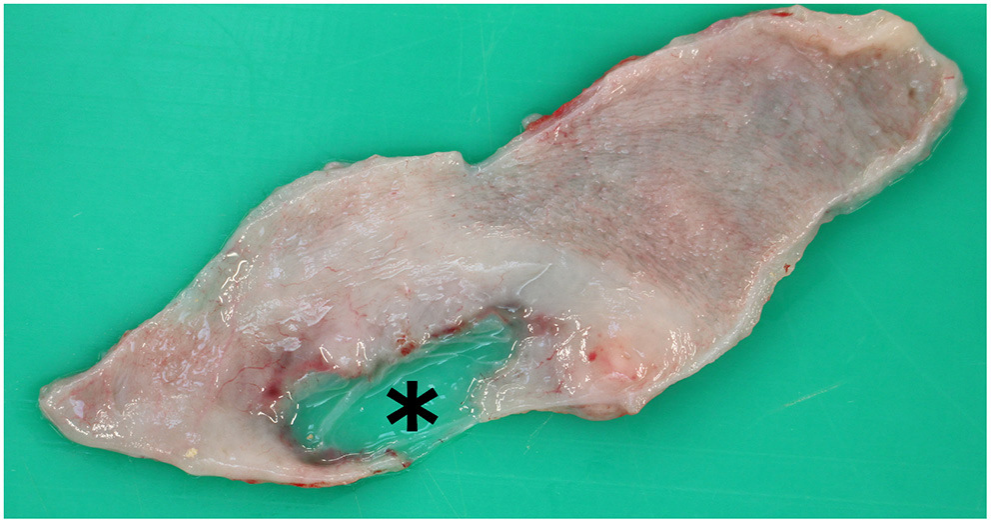
Macroscopic image of the urinary bladder. (Subscript) Local lesion of the bladder wall measuring 1 cm in diameter and only covered by a translucent mucosal membrane (asterisk).

The abdominal cavity was grossly filled with 5 to 6 liters of clear fluid with a urine-like odor, which started to gel after contact with air. A fibrinous peritonitis was highly suspected due to this phenomenon. In the left kidney parenchyma, several small renal calculi were found at the junction between the renal papillae and renal pelvis (nephrolithiasis). The renal pelvis was slightly atrophic. Additionally, the left ureter was mildly enlarged (hydroureter) and filled with a clear fluid interpreted as urine. The urine filled bladder as described above exhibited a malformation of the bladder wall measuring 1 cm in diameter, which was only covered by a translucent mucosal membrane (Figure 1). Furthermore, small amounts of small calculi were detected. The urethra was blocked by a single calculus (2 mm in diameter) 12 cm proximal of the preputium (urolithiasis). The calculus was surrounded by severe hemorrhagic inflammation (hemorrhagic urethritis). The preputial mucous membrane showed several petechiae.

### Histopathologic evaluation

**Figure 2:**
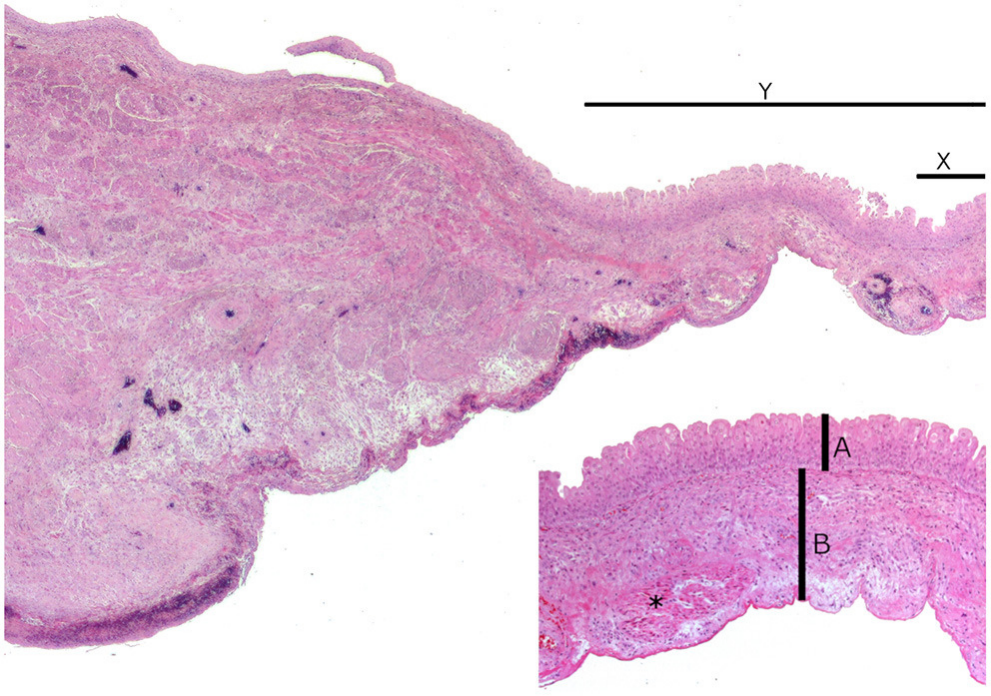
Microscopic image of the urinary bladder. Normal bladder wall on the left and disintegrating ordinary muscle layer on the right side. This defect is flanked by a juvenile granulation tissue, subserosal bleedings, and individual muscle bundles. Normal bladder wall on the left with detaching ordinary muscle layer on the right side. This defect (Y) is flanked by an immature granulation tissue, subserosal hemorrhage, and individual muscle bundles. Inset (X): local lesion only consists of a transitional cell layer (A) with a thin loosely arranged connective tissue covered with mesothelial cells (B). Individual muscle bundles are present (asterisk).

Histologic examinations of the bladder revealed that the defect only consisted of a transitional cell layer with thin loosely textured connective tissue covered with mesothelial cells. At this position, a muscle layer was absent (Figure 2). This defect was flanked by immature granulation tissue, subserosal bleedings, and individual muscle bundles. The individual muscle bundles merged into the normal muscle layer. The regular bladder wall was present with a few bulged activated mesothelial cells which showed serous to hemorrhagic serositis. The left kidney and urethra showed no histologic alterations beyond the macroscopic findings.

## Discussion

### Urolithiasis

To our knowledge, this is the first case of combined urolithiasis, nephrolithiasis as well as a bladder malformation in an alpaca cria. Urolithiasis is a regularly occurring emergency in male small ruminants (1–3), and several working groups have identified predisposing factors (1, 2, 4, 5). In contrast, predisposing factors contributing to the formation of uroliths in llamas and alpacas have only been superficially examined. Surgical intervention in small ruminants (2) as well as in SAC (6) is associated with a higher survival rate compared to non-surgical therapies. Nevertheless, survival rates for small ruminants after surgical intervention vary greatly between studies (1, 2, 5). The study by Riedi et al. (2) showed that 51.6% of the patients survived surgical tube cystostomy after being released from the clinic, whereas higher percentages of survival were reported by Dühlmeier et al. (1) (57.1%) and Ewoldt et al. (5) (76%). Duesterdieck-Zellmer et al. (6) investigated the outcome of combined medical and surgical intervention in SAC with obstructive urolithiasis and reported a survival rate of 65.8% after surgery (6). The surgical procedures analyzed included tube cystotomy, bladder marsupialization, and removal of calculi from the distal ureter, but did not include the therapy of nephroliths. In our case, pathologic examination revealed that a calculus was localized in the distal ureter 12 cm proximal of the prepuce. Performing surgical tube cystotomy or perineal urethrostomy enable a urine outflow from the bladder and reduce the pressure from the urethra. In rams suffering from urolithiasis which were treated by tube cystotomy, it was observed by the authors that in single cases after surgery, also stuck calculi can be released from the urethra (1). Whether this would have been the case in this alpaca remains unclear.

### Nephrolithiasis

Nephroliths are an uncommon finding in ruminants and therefore only a few case reports exist. Ultrasound can be used to visualize nephroliths and the associated degeneration of the corresponding kidney (22, 23). In the reported case, no sonographic examination of the kidneys was performed because primary renal disease was not suspected. In cattle, nephrolithiasis was described in association with previous *Corynebacterium renale* pyelonephritis (22, 23). In the present case, there was neither a history of pyelonephritis nor was there any evidence thereof in the post-mortem examination.

### Azotemia

We suppose that azotemia can be classified as postrenal azotemia due to the obstructions of the urinary tract, urinary accumulation in the renal pelvis, and consequent decreased glomerular filtration. Calculi in both the renal pelvis and the distal ureter may have led to urinary retention, although calculi may also have resulted in obstruction with secondary compression atrophy of the renal pelvis. With the selected method of kidney functional analysis (19, 20), the total renal function can be evaluated simultaneously. However, a differentiated identification of one-sided renal insufficiency is only possible if urine samples from both ureters can be collected *via* retrograde bladder endoscopy. This was not possible in the presented case. To our knowledge, specific reference values for renal functional analysis in SAC have not been published. Nonetheless, these values are expected to differ only slightly from other herbivorous species (19, 20). A case report in cattle showed that unilateral nephrolithiasis might not be associated with azotemia (23), which might be possible as long as there is no obstruction of the distal urinary passages. There is evidence showing that alpacas can live without restrictions after unilateral nephrectomy (24) as other ruminants do (25). In these two previously mentioned case reports, unilateral nephrectomy was not associated with azotemia. Therefore, it is more likely that azotemia was a product of obstructive urolithiasis. A retrospective study investigating obstructive urolithiasis in 270 small ruminants showed that nearly 90% of these animals had azotemia (26). To our knowledge, adequate studies focusing on clinical chemistry in SAC with obstructive urolithiasis have not been performed so far.

### Calculus

It remains questionable which factor promoted the formation of the renal and the urinary calculus. In small ruminants, feeding a diet high in concentrated feed is associated with an increase in urolithiasis due to an imbalanced calcium to phosphate ratio (26). There is a possibility that the increased excretion of phosphate is due to a vitamin D_3_ deficiency. However, the decreased plasma phosphate levels as well as the increased fractional renal phosphate excretion could indicate that increased urinary phosphate concentrations were present in this alpaca. This theory cannot be confirmed because the composition of the calculi was not analyzed. To our knowledge, it has not been investigated whether a deficiency of vitamin D_3_ is a predisposing factor of urolithiasis in alpacas. Under experimental conditions, feeding supraphysiologic amounts of magnesium induced the formation of nephroliths in calves (27). In our case, magnesium intake is very unlikely to be the reason for calculus formation, because magnesium concentration in the plasma was below the lower reference limit. Furthermore, reduced water intake is discussed as a potential predisposition. Whether respective calculus was washed out of the kidney cannot be conclusively clarified.

### Bladder malformation

In such a young animal, a malformation of the urinary tract could also be a cause of reduced urine output, which favors crystallization. Post-mortem examination revealed that the bladder was distended with urine, and exhibited a malformation which was covered with mucosa and was not showing signs of rupture. From the histologic findings, it remains unclear whether the malformation of the bladder was a congenital bladder diverticulum or a pseudo diverticulum. A bladder diverticulum is defined as a bladder mucosa herniation through muscular fibers of the bladder wall (28), whereas a pseudo diverticulum is characterized by a protrusion of the mucosa through gaps in the musculature in the surrounding area of vascular penetration points and is associated with elevated intravesical pressure (29). Bladder diverticula are rarely diagnosed in veterinary medicine (30). As the site of insertion of the Verres needle into the bladder could not be identified with certainty, it remains speculative whether puncturing affected the bladder diverticulum. In this specific case, it remains unclear whether the diverticulum obstructed urinary flow or whether it was purely an incidental finding.

### Anemia

Hematology revealed that the packed cell volume and hemoglobin concentration were decreased. Anemia is a frequent observation in alpacas suffering from chronic diseases (15). Since no further diagnostics concerning anemia were performed, the origin thereof remains unknown. Although this he could not be conclusively clarified, a connection with a coccidia infection cannot be ruled out (18).

## Conclusion

In summary, the diagnosis of urolithiasis or nephrolithiasis should also be considered in young alpacas with urinary dysfunction even if small amounts of urine are excreted. For diagnosis, ultrasonographic investigations not only of the urinary bladder but also of both kidneys should be routinely performed in these cases. As previously reported and as shown in the presented case, conservative therapy for obstructive urolithiasis in SACs has only poor chances of success (6). It remains unclear to what extent nephrolithiasis might have influenced parameters of clinical chemistry, as azotemia could also be due to the distal obstruction of the urethra in the presented case. Furthermore, the cause of the nephrolithic formation can only be speculated. Therefore, pathogenesis and treatment of obstructive urinary diseases in SACs warrant further research.

## Data availability statement

The original contributions presented in the study are included in the article/supplementary material, further inquiries can be directed to the corresponding author/s.

## Ethics statement

Ethical review and approval was not required for the animal study because all data used for this study were collected during clinical treatment, pathological examination, and were obtained to diagnose the clinical case. Written informed consent was obtained from the owners for the participation of their animals in this study.

## Author contributions

JS, MW, DH, and AA diagnosed and treated the clinical case. SK performed the pathologic, histologic examinations, provided the description, figures, discussion of pathology, and histology. JS wrote the manuscript and provided the clinical case description. The study was designed by JS and supervised by MG and MW. All authors read and approved the final manuscript.

## References

[1] Dühlmeier R Zibell G von Altrock A Roth C Schröder C Thies K. Urolithiasis beim kleinen Wiederkäuer—Behandlungsmethoden und klinische Rekonvaleszenz. Tierarztl Prax Ausg G Grosstiere Nutztiere. (2007) 35:175–82. 10.1055/s-0037-1621635

[2] Riedi AK Nathues C Knubben-Schweizer G Nuss K Meylan M. Variables of initial examination and clinical management associated with survival in small ruminants with obstructive urolithiasis. J Vet Intern Med. (2018) 32:2105–14. 10.1111/jvim.15336 30307649 PMC6272034

[3] van Weeren PR Klein WR Voorhout G. Urolithiasis in small ruminants. Vet Q. (1987) 9:76–9. 10.1080/01652176.1987.9694078 3564320

[4] George JW Hird DW George LW. Serum biochemical abnormalities in goats with uroliths: 107 cases (1992-2003). J Am Vet Med Assoc. (2007) 230:101–6. 10.2460/javma.230.1.101 17199500

[5] Ewoldt JM Anderson DE Miesner MD Saville WJ. Short- and long-term outcome and factors predicting survival after surgical tube cystostomy for treatment of obstructive urolithiasis in small ruminants. Vet Surg. (2006) 35:417–22. 10.1111/j.1532-950X.2006.00169.x 16842285

[6] Duesterdieck-Zellmer KF Van Metre DC Cardenas A Cebra CK. Acquired urethral obstruction in New World camelids: 34 cases (1995-2008). Aust Vet J. (2014) 92:313–9. 10.1111/avj.12207 24964920

[7] Ruminants NRCCoNRoS Council NR Ruminants CotNRoS Agriculture Bo Resources N Earth Do. In: Nutrient Requirements of Small Ruminants: Sheep, Goats, Cervids, and New World Camelids. Washington, DC: National Academies Press (2007).

[8] Van Saun R. Urinary Blockage in Llamas Alpacas. (2013). Available online at: https://extension.psu.edu/urinary-blockage-in-llamas-and-alpacas (accessed September 26, 2022).

[9] Roth C Ganter M. Urolithiasis bei einem Lamawallach. Tierarztl Prax Ausg G Grosstiere Nutztiere. (2007) 35:446–52. 10.1055/s-0038-1624026

[10] Jones M Gibbons PM Roussel A Dominguez BJ. Mineral composition of uroliths obtained from sheep and goats with obstructive urolithiasis. J Vet Intern Med. (2017) 31:1473. 10.1111/jvim.14743 28556535 PMC5508333

[11] Hay L. Prevention and treatment of urolithiasis in sheep. In Pract. (1990) 12:87–91. 10.1136/inpract.12.3.87

[12] Cebra C. Chapter 39–disorders of the urinary system. In:Cebra C Anderson DE Tibary A Van Saun RJ Johnson LW, editors. Llama and Alpaca Care. St. Louis: W B. Saunders (2014). p. 464–76.

[13] Güven A Marasli N Kamiloglu N. Nephrolithiasis of sheep in Turkey. Indian Vet J. (2003) 80:409–11.

[14] Hardefeldt LY Textor JA Dart AJ. Renal agenesis in an alpaca cria. Aust Vet J. (2007) 85:185–7. 10.1111/j.1751-0813.2007.00125.x 17470065

[15] Wagener MG Neubert S Punsmann TM Wiegand SB Ganter M. Relationships between Body Condition Score (BCS), FAMACHA©-Score and Haematological Parameters in Alpacas (Vicugna pacos), and Llamas (Lama glama) presented at the veterinary clinic. Animals. (2021) 11:2517. 10.3390/ani11092517 34573483 PMC8469494

[16] Wagener M Grimm L Ganter M. Anaemia in a llama (Lama glama): treatment, regeneration and differential dagnoses. Vet Rec Case Rep. (2018) 6:638. 10.1136/vetreccr-2018-000638

[17] Husakova T Pavlata L Pechova A. Reference values for biochemical parameters in blood serum of young and adult alpacas (Vicugna pacos). Animal. (2014) 8:1448–55. 10.1017/S1751731114001256 24852937

[18] Cebra CK Valentine BA Schlipf JW Bildfell RJ McKenzie E Waitt LH. Eimeria macusaniensis infection in 15 llamas and 34 alpacas. J Am Vet Med Assoc. (2007) 230:94–100. 10.2460/javma.230.1.94 17199499

[19] Brandt KD Glitz E Bickhardt F. Nierenfunktionsanalysen bei Pferden mit Nephropathien Pferdeheilkunde. (1997) 13:335–44. 10.21836/PEM19970404

[20] Bickhardt RD. Clinical studies of kidney function in sheep. I. methods and reference values of healthy animals. Dtsch Tierarztl Wochenschr. (1994) 101:463. 7720545

[21] Judson GJ Feakes A. Vitamin D doses for alpacas (Lama pacos). Aust Vet J. (1999) 77:310–5. 10.1111/j.1751-0813.1999.tb10270.x 10376102

[22] Divers TJ Reef VB Roby KA. Nephrolithiasis resulting in intermittent ureteral obstruction in a cow. Cornell Vet. (1989) 79:143–9. 2647406

[23] Tulleners EP Deem DA Donawick WJ Whitlock RH. Indications of unilateral bovine nephrectomy: a report of four cases. J Am Vet Med Assoc. (1981) 179:696–700. 7341585

[24] Gerspach C Hull BL Rings DM Chew DJ Beamer GL Hubbell JA. Hematuria and transitional cell papilloma of the renal pelvis treated via unilateral nephrectomy in an alpaca. J Am Vet Med Assoc. (2008) 232:1206–9. 10.2460/javma.232.8.1206 18412535

[25] Vogel SR Desrochers A Babkine M Mulon PY Nichols S. Unilateral nephrectomy in 10 cattle. Vet Surg. (2011) 40:233–9. 10.1111/j.1532-950X.2010.00785.x 21223318

[26] Riedi AK Knubben-Schweizer G Meylan M. Clinical findings and diagnostic procedures in 270 small ruminants with obstructive urolithiasis. J Vet Intern Med. (2018) 32:1274–82. 10.1111/jvim.15128 29660779 PMC5980268

[27] Petersson KH Warner RG Kallfelz FA Crosetti CF. Influence of magnesium, water, and sodium chloride on urolithiasis in veal calves. J Dairy Sci. (1988) 71:3369–77. 10.3168/jds.S0022-0302(88)79942-7 3235737

[28] Rasouly HM Lu W. Lower urinary tract development and disease. Wiley Interdiscip Rev Syst Biol Med. (2013) 5:307–42. 10.1002/wsbm.1212 23408557 PMC3627353

[29] McDougal WS Wein AJ Kavoussi LR Partin AW Peters CA. Campbell-Walsh Urology 11th Edition Review E-Book. Amsterdam: Elsevier Health Sciences (2015).

[30] Anson A Strohmayer C Larrinaga JM Iglesias E Almela R Ramírez G. Computed tomographic retrograde positive contrast cystography and computed tomographic excretory urography characterization of a urinary bladder diverticulum in a dog. Vet Radiol Ultrasound. (2019) 60:E66–e70. 10.1111/vru.12591 29333663

